# Adjuvant administration of probiotic effects on sexual function in depressant women undergoing SSRIs treatment: a double-blinded randomized controlled trial

**DOI:** 10.1186/s12888-023-05429-w

**Published:** 2024-01-12

**Authors:** Nazir Hashemi-Mohammadabad, Seyed-Abdolvahab Taghavi, Nicky Lambert, Raana Moshtaghi, Fatemeh Bazarganipour, Mahboubeh Sharifi

**Affiliations:** 1 Department of Psychiatry, School of Medicine, Yasuj University of Medical Sciences, Yasuj, Iran; 2 Medicinal Plants Research Center, Yasuj University of Medical Sciences, Yasuj, Iran; 3 Department of Mental Health and Social Work, Middlesex University, London, England; 4 Social Determinants of Health Research Center, Yasuj University of Medical Sciences, Yasuj, Iran; 5 Department of Midwifery, School of Medicine, Yasuj University of Medical Sciences, Yasuj, Iran

**Keywords:** Probiotic, Sexual function, Depressive disorder, Sexual satisfaction

## Abstract

**Background:**

According to the Institute of Health Metrics and Evaluation’s Global Health Data Exchange (2023) it is estimated that 5% of all adults will experience depressive disorder. Amongst the general loss of pleasure and interest in everyday activities that are symptoms of low mood, reduced sexual desire and sexual dysfunction can be particularly overlooked. Human sexuality is complex, but finding solutions based on scientific evidence to limit the symptoms of depressive disorder and the iatrogenic impact of antidepressant treatment to improve this outcome is an important step in promoting psychological health and general wellbeing.

**Objective:**

The present study aimed is to provide scientific evidence to assess the effect of oral probiotic on sexual function in women with depressive disorder treated with Selective Serotonin Reuptake Inhibitors (SSRIs) in an Iranian population.

**Design:**

This study was a double-blind randomized clinical trial. Eligible women were assigned to lactofem plus SSRIs (*n* = 58) or SSRIs alone (*n* = 54). In group A, SSRI antidepressants were prescribed together with Lactofem, and in group B, SSRI antidepressants were prescribed alone. Lactofem including Lactobacillus acidophilus 2 × 10^9^ cfu/g, Bifidobacterium bifidus 2 × 10^9^ cfu/g, Lactobacillus rutri 2 × 10^9^ cfu/g, Lactobacillus fermentum 2 × 10^9^ cfu/g; capsule weight of 500 mg bio-capsule administered orally and daily. The duration of intervention in two groups was two months. All questionnaires were completed by the patients before and after the intervention. The Female Sexual Function Index (FSFI), Hamilton Depression Rating Scale and Larson's Sexual Satisfaction Questionnaire were used to evaluate sexual function, severity of depressive disorder and sexual satisfaction, respectively.

**Results:**

Based on the results of the present study, there was a statistically significant difference in sexual satisfaction and severity of depressive disorder between the groups before and after the intervention (*P* < 0.05). Also, our findings showed that after eight weeks, the Lactofem plus SSRIs group showed significant improvement in FSFI domains and total scores compared to SSRIs alone group (*P* < 0.05).

**Conclusions:**

The results of the present study show that taking probiotics for eight weeks may improve the severity of depressive disorder, sexual function and sexual satisfaction in depressed women treated with SSRIs.

**Trial registration:**

ClinicalTrials.govidentifier: IRCT20160524028038N14 (19/12/2022)

## Introduction

Depression is a psychological condition characterized by persistent feelings of sadness and a loss of interest in activities [1, 2]. The Diagnostic Statistical Manual of Mental Disorders, Fifth Edition (DSM-5) developed by the American Psychiatric Association categorizes depressive disorders into several subtypes, including disruptive mood dysregulation disorder, major depressive disorder, persistent depressive disorder (dysthymia), premenstrual dysphoric disorder, and depressive disorder due to another medical condition. All these disorders share common symptoms such as sadness, emptiness, or irritability, as well as physical and cognitive changes that significantly impair an individual's ability to function. Unfortunately, due to misconceptions surrounding mental health issues, approximately 60% of individuals with depression do not seek professional help. This reluctance may stem from the fear of societal stigma associated with mental health disorders and the potential negative impact on both personal and professional aspects of their lives [3]. The prevalence of depressive disorder within a twelve-month period is estimated to be around 7%, and this rate varies significantly across different age groups. Notably, females tend to experience rates that are 1.5 to 3 times higher than males. In the United States, approximately 17 million adults are affected by depression; however, these figures are greatly underestimated as a considerable number of individuals have not sought medical attention for their condition [3]. The prevalence of depressive disorder in Iran is moderate and growing which is alarming as it may turn out to be a massive increase in incidence rate of depression in coming years. It constitutes around 35 to 45% of mental health problems in Iran and covers 8% to 20% of population of Iran [4].

Various medicinal and non-medicinal methods are used to treat depressive disorder. The most common drug treatment for depressive disorder is Selective Serotonin Reuptake Inhibitors (SSRIs) antidepressants. Although the prescription of these drugs has vastly increased in recent decades; medication is usually prescribed alongside lifestyle changes, talking therapies and other treatments. In addition, studies have shown that whilst SSRI’s have a lower side effect profile than most antidepressants, they are still associated with adverse effects such as nausea, sweating, tremors, dry mouth, insomnia, constipation, diarrhea, decreased appetite, and sexual dysfunction [5, 6]. The prevalence of sexual dysfunction in these drugs is 50–90%, including delay in orgasm, decreased sexual desire, erectile dysfunction, and pain during intercourse [7–9]. In general, sexual dysfunction in those who suffer from depressive disorder may be considered both a symptom of depressive disorder and a side effect of many antidepressants [10, 11]. When people experience the side effects of antidepressants, they may be tempted to leave the treatment unfinished, which leads to a continuance of low mood and its attendant symptoms [12].

Treatments related to sexual dysfunction following the use of antidepressants include reducing or temporarily halting use, and/or changing medications, as well as using other medications to treat of sexual side effects related to these drugs, such as sildenafil, bupropion, tadalafil, and yohimbine. However, the mentioned drugs have many side effects, including a significant increase in blood pressure [13–16]. Probiotics have been used as a treatment modality for over a century. Probiotics are non-pathogenic microorganisms which are used to treat gastrointestinal and non-gastrointestinal diseases [17]. Many of these microorganisms are part of the normal flora of the human intestine, they live in a symbiotic relationship with the flora of the digestive tract.

Depressive disorder is a disorder marked by continuous psychological stressors which impact the body physically, it interacts with the digestive system through the brain-gut-microbiota axis [18]. It is thought that the therapeutic effects of probiotics also through the central nervous system-gastrointestinal signaling pathway. These bacteria support the production of vitamins, antibiotics and neurotransmitters and their functionality can be altered by taking probiotics and stress factors. An unhealthy microbiome in the gut leads to inflammation, which forms a complete barrier to transmission. Studies have identified sexual dysfunction as common among people with inflammatory arthritis [19, 20] and also noted the connection between the gut and mental health [18, 21, 22].

Currently, one of the newer strategies in the field of mental health treatment is the use of probiotic bacteria. A study aimed at evaluating the effects of probiotics on depression symptoms in humans was conducted by Wallace et al. (2017). The results of this study have shown that probiotics reduce depressive symptoms, but additional double-blind randomized control trials in clinical populations are necessary to further evaluate efficacy [23]. In another study by Zhang et al. (2017)., the “meta-analysis of the use of probiotics to reduce symptoms of depression” compared the use of probiotics with a placebo control group. However, the findings were that there was no significant difference in mood between the treatment and placebo groups after the intervention. In the most recent systematic review conducted in 2023, the effects of adjuvant probiotic administration with first-line treatments for psychiatric illnesses were examined. The findings from this review indicate that all studies on Major Depressive Disorder (MDD) (*n* = 5) and Generalized Anxiety Disorder (GAD) (*n* = 1) demonstrated that adjuvant probiotic or symbiotic treatment was more effective in improving the symptoms of psychiatric illness compared to the first-line treatment alone or with a placebo [24]. However, a previous study conducted by Rudzki et al. in 2019 did not observe any significant effects on symptom severity in MDD patients who received adjuvant probiotic therapy, as measured by the HAM-D, Symptom Checklist-90 (SCL-90), and Perceived Stress Scale-10 (PSS10). Nonetheless, this study did find a positive correlation between adjuvant probiotic treatment and increased cognitive function [25]. To the best of our understanding, there is a lack of research examining the impact of probiotic supplementation on depressive disorder depressive disorder symptoms, sexual satisfaction, and function in individuals with depressive disorder who are also taking pharmacological antidepressants. Only one study has been conducted on the effects of probiotics on sexual function in individuals with Polycystic Ovary Syndrome (PCOS) [26]. Given the association between increased oxidative events and inflammatory cytokines in PCOS same as depressive disorder, it is plausible that altering the physiological equilibrium of microorganisms in the gut microbiome contributes to the pathophysiology of PCOS. Therefore, utilizing probiotics may help restore this balance. Differences between studies regarding probiotic dose, bacterial strains, and strain composition limit the comparability of these and other current clinical trials. Considering that widespread nature of depressive disorder and the lack of studies on the impact of probiotics on the sexual function, the present study is key in providing scientific evidence as the first RCT to assess the effect of oral probiotic on sexual function in women with depressive disorder treated with SSRIs in an Iranian population. It should be noted considering the observed sex differences in microbiome composition [27] that may affect gut-brain axis activity and response to probiotics, our study focused to female population.

## Methods

### Participants

This study was a randomized clinical trial undertaken in women with depressive disorder treated with anti-depressants attending in outpatient Shahid Mofatteh clinic in Yasuj, Iran. This study was a randomized controlled trial registered at the Iranian Registry of Clinical Trials, IRCT20160524028038N14, prospectively registered from 19/12/2022. In addition, the study was approved by the Yasuj University of Medical Sciences Ethics Committee with ethical code: IR.YUMS.REC.1401.078. An information sheet was offered to all women and informed written consent was obtained. The participants were invited to join the study during 2023 and all participants were aware that they could withdraw any time during the trial.

### Recruitment of participants

#### Inclusion criteria

Age 18–45 years; being able to read and write; scoring higher than 20 on the Hamilton Depression Rating Scale (HDRS) indicating at least moderate severity of depressive disorder; being treated for at least four weeks with antidepressants; having sex at least once a month; having an emotionally satisfying relationship with a stable and sexually active partner for at least one year. Diagnosis of depressive disorder was verified by a specialist in psychiatry prior to inclusion.

#### Exclusion criteria

Disinclination to join or remain within the study, having speech or hearing problems, addiction to drugs and alcohol in patient or her partner, having declared gynecological disease in patient and/or sexual disorders with organic origin, having sexual problems before taking antidepressants, having symptoms of psychosis, recent close bereavement (during the last six months), known sexual problems in partner, current pregnancy and breastfeeding, suffering from internal diseases such as cardiovascular, thyroid, arthritis, diabetes, epilepsy, kidney infection, taking birth control pills or any other medication that can affect sexual function, such as antihypertensives drugs, antihistamine H2 blockers, barbiturates.

The sample size was considered based on Moludi et al. (2019) [28] using the following formula; as a minimum 52 persons were assessed for each group.

$$N=\frac{{\left({Z}_{1-\frac{\alpha }{2}}+{Z}_{1-\beta }\right)}^{2}\left({S}_{1}^{2}+{S}_{2}^{2}\right)}{{\Delta }^{2}}$$

$$1-\alpha =\%95 \beta =\%80$$

"Z is the value from the table of probabilities of the standard normal distribution for the desired " "confidence level "("e.g.,Z = 1.96 for 95% confidence")

$${S}_{1}=3.6{S}_{2}=5.9 {\Delta }^{2}=7.29$$

### Randomization, hidden distribution, and blindness

Allocation to Lactofem plus SSRIs (Group A) or SSRIs alone (Group B) was done by “block” randomization method. The randomization tool was statistical software; and the study was double-blind using envelopes in the package with neither the participants nor physicians knowing either the allocation or group assignment.

### Study design

After obtaining written consent from eligible participants to enter the study, the subjects were evaluated for two months: Time period 1 (before the intervention): with their depressive disorder severity measured using Hamilton Depression Rating Scale, their sexual function by FSFI, sexual satisfaction by Larson sexual satisfaction questionnaire. After completing the questionnaires, the subjects were divided into two intervention and control groups.

#### Group A

Had SSRIs antidepressants (fluoxetine, sertraline, fluvoxamine, citalopram, escitalopram, paroxetine) plus Lactofem prescribed. Lactofem capsule (including Lactobacillus acidophilus 2 × 10^9^ cfu/g, Bifidobacterium bifidus 2 × 10^9^ cfu/g, Lactobacillus rutri 2 × 10^9^ cfu/g, Lactobacillus fermentum 2 × 10^9^ cfu/g; capsule weight of 500 mg bio-capsule) made by Iranian zist-takhmir Company administered orally and daily for two months.

#### Group B

Had only SSRIs antidepressants (fluoxetine, sertraline, fluvoxamine, citalopram, escitalopram, paroxetine) prescribed.

Duration of intervention in two groups were two months.

### Outcome measure

1. Demographic data was collected including age, formal education, occupation, BMI, reproductive history, and type of anti-depressant used.
2. Sexual function: taken as impairment of sexual desire and arousal, dyspareunia and orgasmic dysfunction. The questionnaire of Female sexual function index (FSFI) was used in the present study to evaluate sexual function. FSFI was developed by Rosen et al. (2000) to evaluate sexual function over a four-week period. The 19 items questionnaire measures female sexual function in six independent dimensions experiencing sexual desire (four items), arousal (four items), lubrication (four items), orgasm (three items), satisfaction (three items) and pain (three items). The answers are rated from zero to five where a higher score indicates more satisfactory sexual function [29]. The reliability and validity of the questionnaire are approved in Iran [30].
3. Sexual satisfaction: to measure this item, we used the Larson questionnaire which consists of 25 questions. The responses are rated as never to always (5) with the higher score indicating greater levels of sexual satisfaction [31]. The reliability and validity of the questionnaire have been acknowledged [32].
4. Measurement of depressive disorder severity: was accomplished in this study using the *Hamilton Depression Rating Scale*; we used the Persian version with 21 items; including psychometric indices. The rating scale explores symptoms related to depression, including low mood, suicidality, irritability, tension, loss of appetite, loss of interests, and somatic symptoms. Answers were given on different rating scales ranging from 3-, 4- or 5-point ratings: (e.g., “insomnia early”: 0 = no difficulty falling asleep; 1 = complains of occasional difficulty falling asleep i.e., more than 0.5 h; 2 = complains of nightly difficulty falling asleep), with higher scores reflecting more marked depressive symptoms [33]. The reliability and validity of the questionnaire are approved in Iran [34].
5. Side effects, i.e., fever, itching, diarrhea, vomiting, or other gastrointestinal symptoms were recorded.

### Statistics

Data analysis was done using descriptive statistics (frequency, percent, mean ± SD) and a comparison of these data was performed by x^2^ and the independent t-test We used descriptive statistics as well as the Kolmogorov–Smirnov test to analyze the distribution of data. The statistical program for social sciences (SPSS, version 21; SPSS, Chicago, IL). *P* values were set at 0.05 for all analyses. In this study, sexual function was considered as the primary outcome and depressive disorder severity and sexual satisfaction considered as the secondary outcome. There were no missing values. Therefore, no missing imputation technique was used. This manuscript is in accordance with the PRISMA guidelines for reporting randomized trials.

## Result

### Sample characteristics

**Fig. 1:**
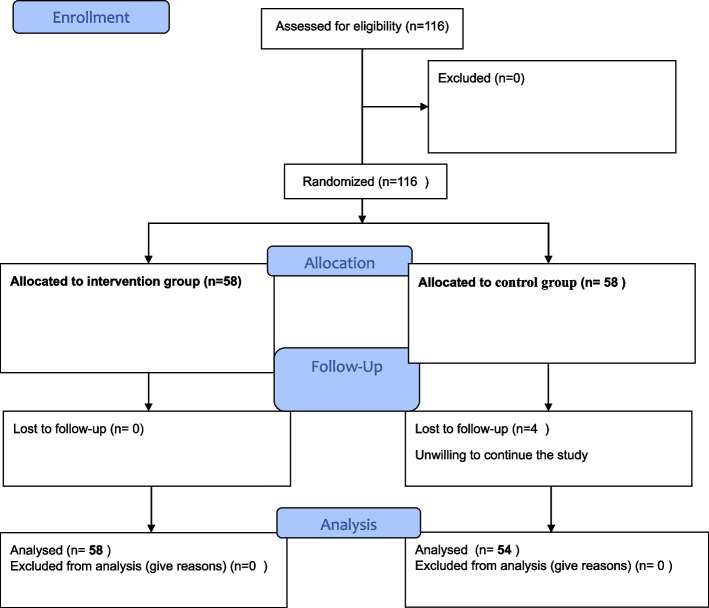
Study flow diagram

A total of 116 patients were examined and were randomly divided into two groups (58 patients in each group). Lastly, 112 patients finalized the follow-up (58 and 54 patients in the probiotic and control groups, respectively). The process of patient allocation is shown in Fig. 1. According to Table 1, there was no significant difference in socio-demographic characteristics between the studied groups (*P* < 0.05).

**Table 1:** Socio-demographic characteristic between groups

Group		Probiotic group *N* = 58	Control group *N* = 54	*P* value
Variable				
Age (year)^a^		35.03 ± 6.75	35.11 ± 6.88	0.95
Education^b^	Secondary school	23 (39.7)	16 (29.6)	0.42
	High school / diploma	24 (41.4)	22 (40.7)	
	Bachelor's degree and higher	11 (19)	16 (29.6)	
Occupation^b^	Employee	7 (12.1)	14 (25.9)	0.04
	Housewife	51 (87.9)	40 (74.1)	
BMI (kg/m^2^)^a^		27.2 ± 4.22	26.03 ± 3.7	0.12
Gravid^a^		3.22 ± 0.89	2.88 ± 0.69	0.32
Parity^a^		2.63 ± 0.69	2.58 ± 0.48	0.86
Medication^b^	Cytalopram	35 (60.3)	43 (79.6)	0.07
	Floxetine	2 (12.1)	5 (9.3)	
	Paroxine	3 (5.2)	2 (2.77)	
	Ezipam	0 (0)	2 (2.77)	
	Certraline	5 (8.6)	2 (2.77)	
	Escitral	8 (13.8)	2 (2.77)	

^a^Mean ± SD; independent t test

^b^n (%); x^2^

^£^*BMI* Body mass index

### Comparison of sum score of sexual function between group

The findings of Table 2 show that sexual desire, arousal, lubrication, orgasm, satisfaction and pain dimensions and sum score of sexual function were significantly better in Lactofem compared with SSRIs group (*P* < 0.05).

**Table 2:** Comparison female sexual function index scores between groups

Group		Probiotic group *N* = 58	Control group *N* = 54	*P* value^**^
Variable^a^				
Desire	Before	2.63 ± 1.11	2.78 ± 1.23	0.58
	After	5.37 ± 0.5	3.73 ± 0.99	< 0.001
	*P* value^#^	< 0.001	0.05	
Arousal	Before	2.53 ± 1.11	2.02 ± 1.32	0.21
	After	5.68 ± 0.38	3.84 ± 0.75	< 0.001
	*P* value^#^	< 0.001	0.05	
Lubrication	Before	3.54 ± 1.37	3.68 ± 1.15	0.15
	After	5.79 ± 0.34	4.36 ± 0.72	< 0.001
	*P* value^#^	< 0.001	0.05	
Orgasm	Before	3.37 ± 1.34	3.51 ± 1.12	0.53
	After	5.7 ± 0.3	4.64 ± 0.71	0.01
	*P* value^#^	< 0.001	0.05	
Satisfaction	Before	3.95 ± 1.28	3.65 ± 0.83	0.18
	After	5.76 ± 0.4	4.93 ± 0.75	< 0.001
	*P* value^#^	< 0.001	0.05	
Pain	Before	4.17 ± 1.29	4.6 ± 0.8	0.52
	After	5.62 ± 0.45	4.98 ± 1.07	0.05
	*P* value^#^	< 0.001)	0.05	
Total score	Before	20.16 ± 5.55	21.48 ± 5.71	0.34
	After	33.85 ± 1.63	25.03 ± 4.36	< 0.001
	*P* value^#^	< 0.001	0.05	

^a^Mean ± SD

^**^Independent t test

^#^Paired t test

Also, there was a statistically significant difference in the Lactofem (*p* < 0.05) and SSRIs (*p* < 0.05) groups the intervention in all of sexual dimensions before and after of Lactofem and SSRIs treatment alone received.

### Comparison of sum score of depressive disorder between group

The findings of Table 3 show that the severity of depressive symptoms was significantly improved in Lactofem compared with SSRIs group (*P* < 0.05). However, the result of paired t test indicated a statistically significant difference in the Lactofem (*p* < 0.05) and SSRIs (*p* < 0.05) groups in depressive disorder severity before and after Lactofem and receiving SSRIs treatment alone.

**Table 3:** Comparison depressive disorder severity between groups

Group		Probiotic group *N* = 58	Control group *N* = 54	*P* value^**^
Variable^a^				
Hamilton Depression Rating Scale score	Before	12.57 ± 2.63	11.77 ± 2.24	0.08
	After	1.43 ± 0.68	3.29 ± 0.51	0.001
	*P* value^#^	0.001	0.001	

^a^Mean ± SD

^**^Independent t test

^#^Paired t test

### Comparison of sum score of sexual satisfaction between group

Based on Table 4, the results of independent t test have shown that the level of sexual satisfaction reported were significantly improved in probiotic group compared with the SSRIs group (*p* < 0.05). Also, the result of paired t test has shown that there was a statistically significant difference in the Lactofem (*p* < 0.05) and SSRIs (*p* < 0.05) groups in sexual satisfactions before and after of Lactofem and receiving SSRIs treatment alone.

**Table 4:** Comparison marital satisfaction between groups

Groups		Probiotic group *N* = 58	Control group *N* = 54	*P* value^**^
Variable^a^				
Marital satisfaction^b^	Before	73.59 ± 7.88	72.55 ± 7.36	0.93
	After	80.23 ± 4.76	76.61 ± 5.81	0.05
	*P* value	< 0.001	0.05	

^a^Mean ± SD

^**^Independent t test

^b^Paired t test

### Side effects

No side events were reported in either group.

## Discussion

The experience of depressive disorder is associated with a significant decrease in quality of life, reduced productivity, diminished overall health, and potentially untimely death. Treatment-related side effects often affect concordance with medications and may lead to treatment discontinuation. Medication nonadherence is a key issue as it can result in an incomplete response to or no improvement in depressive symptoms. In addition, to which sexual dysfunction is a commonly reported side effect either from unmanaged depressive disorder or though iatrogenic side effects after taking prescribed medication. This again reduces the likelihood of adherence to prescribed medication leading to recovery.

To our knowledge, this study is the first to evaluate the effects of probiotic supplementation on depressive symptoms, sexual satisfaction, and function in a sample of women with depressive disorder, as an adjunct to pharmacological antidepressants.

In this randomized clinical trial with female subjects, probiotic supplementation for 8 weeks compared to SSRIs alone led to a greater reduction in depressive disorder scores, improved sexual function, and sexual satisfaction. As no other studies were found on the effect of probiotic supplementation on depressive symptoms, sexual satisfaction, and function in a depressed sample the results of some studies related to investigating the effect of probiotics on other conditions were used to compare with the findings of the present study.

A single study has been conducted on the effect of probiotics on sexual function in the Polycystic ovary syndrome [26] population. Considering the role of increased oxidative events and inflammatory cytokines in PCOS, it is possible that changing the physiological balance between microorganisms in the gut microbiome plays a role in the pathophysiology of PCOS, and the use of probiotics may restore this balance. Previous findings have shown that eight weeks of probiotic consumption may decrease the LH/FSH ratio and improve chemical and clinical pregnancy rates, sexual function, and body satisfaction in women with PCOS. In our study, the improvement of sexual function after probiotic adjuvant during antidepressant treatment seems to parallel the improvement of depressive symptoms.

Depressive disorder is considered a disorder that involves not only a traditional monoamine deficiency [35], but also a persistent low-grade inflammation [36]. People with depressive disorder have shown different levels of increased inflammatory markers compared to people who are not depressed [36–38]. Gut microbiota is considered as a virtual endocrine organ that has a two-way communication with the central nervous system through the microbiome-gut-brain axis [39, 40]. Probiotics can contribute to depressive disorder directly or through the influence of other gut microbes, by inhibiting gut and systemic inflammation and activating the microbiome-gut-brain axis [41–43]. Probiotics directly protect the intestinal barrier. limiting the overgrowth of bacteria in the small intestine; and the reduction of systemic cytokines, oxidative stress and inflammatory markers. and increases microbial production of GABA and other neurotransmitters.

Benton et al. (2007) conducted a double-blind randomized control trial of 124 people. They found that after 20 days of consumption of a probiotic milk drink, although it had no measurable effect on mood states, participants' self-reported mood ratings rose [44]. Rao et al. conducted a study with 35 patients with chronic fatigue syndrome who were randomized to receive probiotics or placebo three times a day for two months. Symptoms of depressive disorder and anxiety were evaluated with Beck Depression Inventory and Beck Anxiety Inventory [45] before and after the intervention and the results showed that while probiotic consumption improved anxiety scores, it had no effect on depressive disorder symptoms. Likewise, Yang Chul Chang et al. conducted a 12-week double-blind randomized controlled trial with healthy subjects aged 60–75 years and found no significant improvement on the Geriatric depression scale after treatment with probiotic fermented milk [46]. Nevertheless, Masoudi et al. (2011) in research on healthy humans found that taking probiotic supplements reduced physical scores, depression, and anger hostility in the Hopkins Symptom Checklist, as well as reducing Hospital Anxiety and Depression Scale scores [47].

Steenbergen et al. (2015) conducted a randomized trial that evaluated the effects of probiotics on cognitive reactivity to sad mood using the Leiden index of depression sensitivity. Forty healthy young adults took a probiotic supplement or a placebo for four weeks and found that taking a multispecies probiotic formula significantly reduced the overall cognitive response to depression, particularly aggressive and ruminative thoughts [48]. Akkasheh et al. (2016) designed a randomized, double-blind, placebo-controlled trial with 40 patients with depressive disorder. Participants took a probiotic supplement or a placebo for 8 weeks and were assessed using the BDI at baseline and post-treatment. Results showed that probiotic supplementation significantly reduced BDI scores, indicating overall improvement in symptoms including mood [49]. Interestingly, the absence of side effects and side effects associated with probiotics in our study suggests that its use was well tolerated and safe in this population.

However, patients in the SSRIs group also experienced improvements in depressive disorder, sexual function, and satisfaction after 8 weeks of SSRIs treatment compared to before antidepressant treatment. It has been found in previous studies that sexual performance in women improves during treatment with antidepressants in parallel with improvement in depressive symptoms, whereas the opposite may be true for men [50, 51]. SSRIs are often considered first-line drug therapy for moderate to severe depression. However, treatment with SSRIs further impairs sexual function and causes immediate sexual dysfunction in 80% of patients with depression [52–55]. Thus, a better response to antidepressant treatment may predict improved sexual performance, whereas a worse response or no response to treatment may predict unchanged or worsening sexual performance. Other research shows that while sexual function is initially more severely impacted in women than in men, it often improves during treatment with antidepressants. The same is true for men [51, 56]. Findings of an overall positive effect of SSRI treatment on sexual performance are supported in other studies [57–59].

The topic of this current research could be crucial for many people who are seeking relief from the symptoms of depressive disorder. Antidepressants are often associated with side effects of a magnitude that cause a significant proportion of people to discontinue their medication. In the above studies, the duration of intervention varies widely, the amount and strains of probiotics, inconsistency in the definition of depressive disorder in different studies. While typical clinical antidepressant treatments last approximately 6 weeks [60], the ideal length of time to observe specific effects of probiotics is unknown. Further research is recommended to determine the effectiveness of probiotics to reduce depressive disorder symptoms and sexual function, as well as the ideal duration of treatment, dose and strain of probiotics to achieve effectiveness in terms of mental health. It is also suggested to classify response to treatment based on gender and age. This is important given the observed sex differences in microbiome composition [61] that may affect gut-brain axis activity and response to probiotics.

### Strengths and limitations

The current study also has strengths, including the use of a gold standard RCT study design with strict inclusion criteria at its baseline. In addition, this questionnaire is based on the previously ratified tools used. A sufficient sample size has been used, which increases the power of the statistical analysis. However, there are several limitations in the present study that must be acknowledged. First, the imported samples were all from the university referral clinic. Also, all patients in this study were married in Iran due to cultural reasons. Moreover, patients may have benefited during the study from psychotherapy and counseling intervention, often used without prescription to prevent or alleviate symptoms at onset. Also, the effectiveness of non-surgical brain stimulation techniques e.g., repetitive transcranial magnetic stimulation (rTMS), and transcranial direct current stimulation (tDCS) and Electroconvulsive therapy (ECT) as alternative or add-on treatments of depressive disorders has been proven in previous studies [61]. These interventions are very effective treatments that can mask the investigated symptoms, but this issue was not measured in the present study. Although it seems that due to the random allocation of patients, this limitation existed randomly in both groups and did not have a significant effect on the results, however, it is recommended to addressed these limitations in future studies. Therefore, the results of the present study should be interpreted with caution. Third, the study did not include a placebo arm. Therefore, we cannot attribute the effects on sexual performance, improvement in depressive disorder, and sexual satisfaction to probiotic treatment per se. Fourth, the patients were younger, which is associated with fewer side effects of serotonergic antidepressant treatment. We cannot rule out that the age of the population acted as a protective factor against sexual side effects and therefore the effect of reducing depressive symptoms on sexual performance is more pronounced. Finally, there was no post-intervention follow-up period. A post-intervention washout period can provide insight into how long clinical effects last or whether long-term use of probiotics is required to maintain these effects.

## Conclusion

In conclusion, taking into account the limitations mentioned above, the results of the current first RCT study suggest that eight weeks of probiotic consumption may improve depressive disorder severity, sexual function and sexual satisfaction in SSRI-treated women.

## Data Availability

The primary data for this study is available from the authors (NH.M) on direct request.

## Abbreviations

SSRIs: Selective Serotonin Reuptake Inhibitors
